# SYMPTOM OVERLAP BETWEEN SLEEP DISORDER AND DEPRESSIVE SYMPTOMS

**DOI:** 10.1093/geroni/igae098.3728

**Published:** 2024-12-31

**Authors:** Abolade Oladimeji, Kari O’Donnell, Douglas Gunzler, Kristen Berg, Douglas Einstadter, Elizabeth Pfoh, Jarrod Dalton, Adam Perzynski

**Affiliations:** Case Western Reserve University, cleveland, Ohio, United States; MetroHealth, Cleveland, Ohio, United States; CWRU, Cleveland, Ohio, United States; CWRU and MetroHealth, Cleveland, Ohio, United States; MetroHealth, Cleveland, Ohio, United States; Cleveland Clinic, Cleveland, Ohio, United States; Cleveland Clinic, Cleveland, Ohio, United States; MetroHealth, Cleveland, Ohio, United States

## Abstract

**Introduction:**

The PHQ-9, a common screening tool for depression, asks about sleep and fatigue. An under-recognized concern is the overlap between sleep and fatigue depressive symptoms and sleep disturbances. We investigated whether elevated depression screening scores were due to sleep disorders rather than depression itself in older adults.

**Methods:**

The study cohort included 2,257 adults (65+) with at least two outpatient primary care visits and at least one Medicare Annual Wellness Visit, who initially screened positive on the PHQ-2 (≥2) and completed the PHQ-9. A multiple indicator multiple cause (MIMIC) analysis was used to examine differential item functioning of the sleep and fatigue items of the PHQ-9, attributable to sleep disorders, based on diagnosis codes.

**Results:**

The MIMIC analysis revealed a positive association between sleep disorders and depression (β = 0.19, p < 0.001), and a small positive effect on the “feeling tired” fatigue item of the PHQ-9 (β = 0.06, p < 0.001). No significant relationship was observed between sleep disorders and the PHQ-9 “sleep” item. The model exhibited a good fit, with RMSEA=0.040 (90% CI: 0.034-0.047), CFI=0.99, TLI=0.99, and SRMR=0.04.

**Discussion:**

Sleep disorders and depressive symptoms are associated but no meaningful differential item functioning was detected in this sample of older adults. The PHQ-9 is an adequate screening tool for depression in older adults with sleep disorders.

